# Increased Expression and Amplification of *bla*_KPC-2_ Contributes to Resistance to Ceftazidime/Avibactam in a Sequence Type 11 Carbapenem-Resistant Klebsiella pneumoniae Strain

**DOI:** 10.1128/spectrum.00955-22

**Published:** 2022-07-28

**Authors:** Xinhui Li, Jisheng Zhang, Chengru Yang, Jie Li, Jianmin Wang, Wan Huang, Lingyi Zeng, Xushan Liang, Wenzhang Long, Xiaoli Zhang

**Affiliations:** a Department of Microbiology, Yongchuan Hospital of Chongqing Medical University, Chongqing, China; b Department of Microbiology, The First Affiliated Hospital of Jiamusi University, Jiamusi, China; c Department of Microbiology, Jiaxing Maternity and Child Health Care Hospital, Jiaxing, China; Emory University School of Medicine

**Keywords:** ceftazidime-avibactam resistance, carbapenem-resistant *Klebsiella pneumoniae*, *bla*_KPC-2_, IS*26*

## Abstract

Ceftazidime/avibactam (CAZ/AVI) is regarded as an effective alternative antibiotic for the clinical treatment of Klebsiella pneumoniae carbapenemase (KPC)-producing isolates. As resistance has been reported in some strains, it is critical to understand the key mechanisms contributing to the acquired resistance to CAZ/AVI. From January 2018 to April 2020, 127 KPC-producing carbapenem-resistant Klebsiella pneumoniae strains (CRKPs) were isolated at a university hospital in Chongqing, China, and 25 strains showed reduced susceptibility to CAZ/AVI. All reduced-susceptibility CRKPs were deficient in Ompk35 and Ompk36 porins, and 24 strains had a premature termination at amino acid position 63 in Ompk35 and 134 to 135 glycine and aspartic acid (GD) insertion in OmpK36, while the *bla*_KPC-2_ expression level showed no significant difference compared to that of strain BAA-1705. Four reduced-susceptibility strains evolved resistance under selective pressure of CAZ/AVI with the *bla*_KPC-2_ expression level increased, and two of these strains had mutations in the Ω-loop. The study found a strain of CRKP55 with changes in the resistance phenotype during conjugation, evolving from reduced sensitivity to high-level resistance to CAZ/AVI. Through plasmid sequencing and reverse transcription-quantitative PCR, it was speculated that insertion sequence (IS)*26*-mediated *bla*_KPC-2_ gene amplification caused the MIC value change in the conjugant JKP55. Our findings illustrated the potential of CAZ/AVI resistance under antibiotic stress and demonstrated that IS*26* may mediate *bla*_KPC-2_ replication transposition, leading to high-level resistance during horizontal gene transfer. Investigation of CAZ/AVI resistance mechanisms may offer a unique opportunity to study the horizontal evolutionary trajectories of K. pneumoniae high-risk clones.

**IMPORTANCE** Klebsiella pneumoniae carbapenemase (KPC) production is the most common mechanism of K. pneumoniae resistance to carbapenems in China. Currently, CAZ/AVI is considered a potential alternative therapeutic option for infections caused by these isolates. However, there have been increasing reports of resistant or reduced-sensitivity strains since the approval of this agent. In this study, resistance to CAZ/AVI was induced under drug-selective pressure and was caused by *bla*_KPC-2_ overexpression and/or substitutions in the Ω-loop of KPC. Additionally, it was demonstrated that a conjugative plasmid carrying *bla*_KPC-2_ could transfer horizontally between species, and perhaps, IS*26*-derived tandem amplification of *bla*_KPC-2_ during this period led to high-level resistance to CAZ/AVI. Our research suggests that IS*26*-mediated resistance evolution may have important implications in guiding clinical antibiotic use.

## INTRODUCTION

*Enterobacterales*, one of the most common sources of community-acquired and hospital-acquired infections, are easily transmitted between humans and have a tendency to acquire resistance genes (1). With the long-term and widespread use of antibiotics, the proportion of carbapenem-resistant *Enterobacterales* (CRE) has gradually increased (2). Klebsiella pneumoniae carbapenemase (KPC)-producing Klebsiella pneumoniae (KPC-KP), which have spread extensively throughout the world, are an important cause of nosocomial infections, especially urinary tract, respiratory tract, and bloodstream-associated infections (3, 4). To meet the medical need for new treatment options, several new antibiotics have been developed and registered recently, including β-lactamase inhibitor combinations, ceftazidime/avibactam (CAZ/AVI), meropenem (MEM)/vaborbactam, and imipenem/relebactam.

CAZ/AVI is a novel β-lactam/β-lactamase inhibitor combination that was approved by the Food and Drug Administration in 2015. This combination has great potency against CREs with class A and C β-lactamases, but not metallo-β-lactamases (MBL) (5). In 2015, Livermore et al. (6) used single-step and multistep selections to induce resistance to CAZ/AVI, which proved that the Asp179Tyr substitution in KPC-3 led to resistance. In the same year, the first CAZ/AVI-resistant strain was reported in a patient without CAZ/AVI exposure history (7). Since then, sporadic reports of CAZ/AVI resistance have rapidly increased, notably in KPC producers. Acquired resistance to CAZ/AVI has been reported to involve several mechanisms, including increased expression of *bla*_KPC-2_ and specific mutations in genes encoding carbapenemases, such as substitutions within the Ω-loop of KPC (positions 165 to 179) and AmpC (8), which decrease the binding of AVI. In addition, membrane impermeability due to porin mutations has been shown to contribute to the development of resistance in knockout experiments (9).

To understand the epidemiological characteristics of CAZ/AVI resistance in patients without CAZ/AVI exposure history in our hospital, the mechanisms of resistance to CAZ/AVI, and the changes in resistance phenotype in the presence of antibiotic pressure, we investigated the CAZ/AVI resistance situation from 2018 to 2020. Here, we report the genetic and phenotypic characterization of the reduced susceptibility to CAZ/AVI in the KPC-KP strain after conjugation.

## RESULTS

### Homology analysis, antibiotic resistances, and susceptibility test.

**FIG 1:**
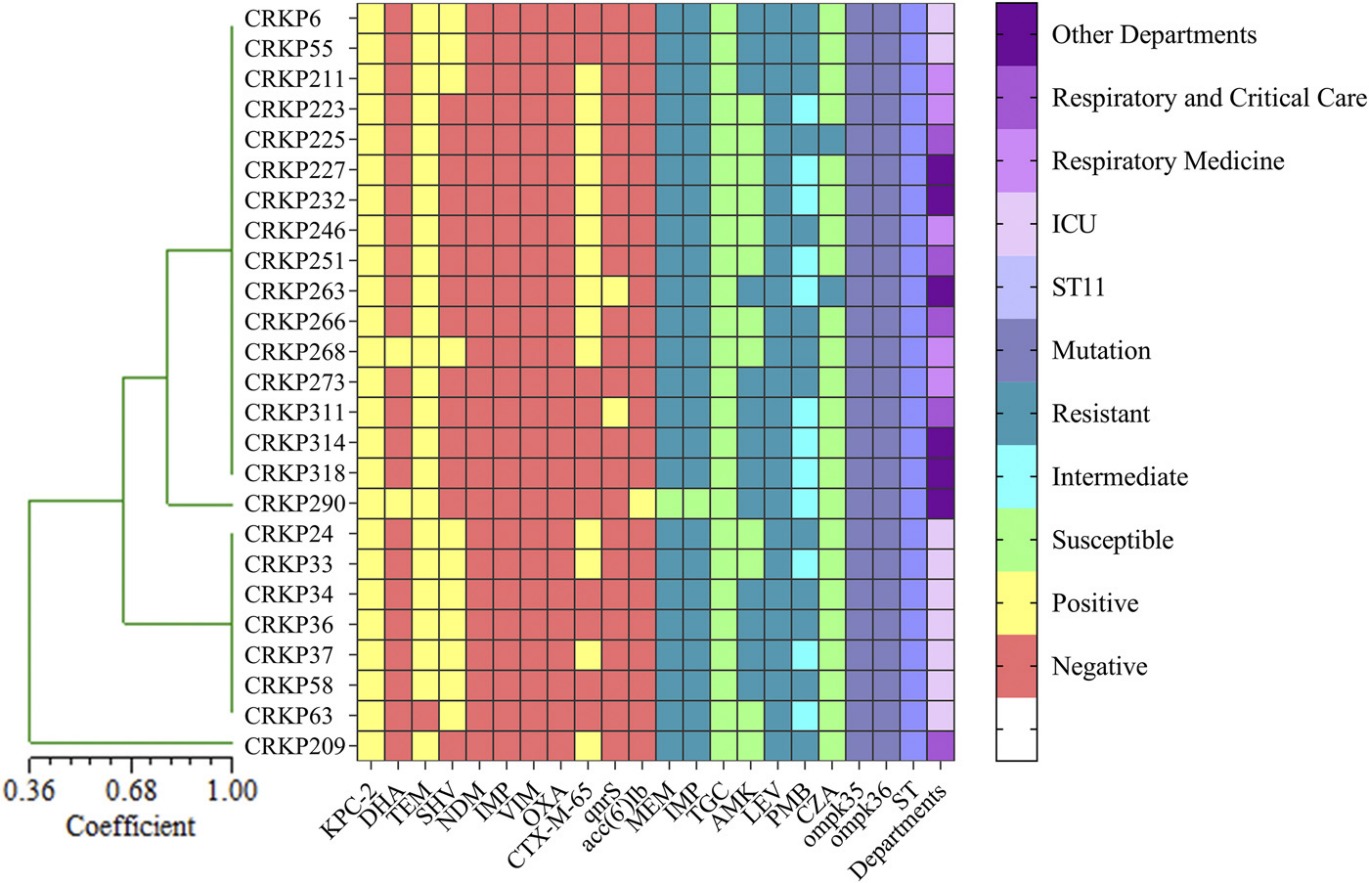
Cluster analysis and characteristics of selected strains (include 23 reduced-susceptibility strains and 2 CAZ/AVI-resistant strains). There was a premature stop codon on amino acid 63 in OmpK35 in all isolates, except for CRKP290, which had premature stop codon on amino acid 82. As for OmpK36, there was a 135 to 136 GD insertion in all strains, except for CRKP290, which had frameshift mutations at amino acid 184. “Other departments” includes infection, neurology, rehabilitation, NICU, and geriatrics.

A total of 16 strains resistant to CAZ/AVI were observed, including 14 strains producing MBL or both *bla*_NDM_ and *bla*_KPC-2_ and two strains only producing *bla*_KPC-2_. In addition, 23 strains producing *bla*_KPC-2_ showed reduced susceptibility to CAZ/AVI, with MIC between 4/4 mg/L to 8/4 mg/L. All 25 CRKPs belonged to sequence type 11 (ST11), and ERIC image analysis divided them into four major clusters (Fig. 1). To explore the relationship between *bla*_KPC-2_ and reduced susceptibility to CAZ/AVI, 25 strains were subjected to further analyses.

### *In vitro* induction test.

The MIC values of the strains with reduced susceptibility showed different degrees of increase under the double-augmented concentration of CAZ/AVI. YKP63 and YKP246 had 4-fold increases in MIC, and YKP223 had a 16-fold increase in MIC, the value of which increased from 4/4 mg/L to 128 mg/L (Table 1).

**TABLE 1:** MIC changes of antibiotics and resistance characteristics during the induction experiment^*a*^

Isolate	CZA/AVI MIC (mg/L)		MEM MIC (mg/L)		Imipenem MIC (mg/L)		KPC gene	
	Original	After induction	Original	After induction	Original	After induction	Original	After induction
KP63	4/4	16/4 (I)	256	512 (I)	256	256	WT	WT
KP209	4/4	64/4 (I)	512	512	32	64 (I)	WT	R164S
KP223	4/4	128/4 (I)	512	256 (D)	128	128	WT	WT
KP246	8/4	32/4 (I)	256	32 (D)	32	8 (D)	WT	D176N

a I, increase; D, decrease. All four strains had increased an MIC value in CAZ/AVI compared to original strains.

### Conjugation test and fitness cost assessment.

Six strains were identified as conjugants using Vitek-2 and 16S rRNA. The success rate was 24% (6/25 patients). All conjugants were resistant to both MEM and rifampicin, and successful transfer of the *bla*_KPC-2_ gene was confirmed using PCR. Compared with that of the original donor CRKP55, the MIC value of carbapenems (MEM) in the conjugant JKP55 was significantly decreased, but the MIC value of CAZ/AVI increased from 4/4 mg/L to 256/4 mg/L (see Table S1 in the supplemental material). As shown in Fig. S1, no significant difference (*P* > 0.05) in growth was observed between the two isolates.

### Mutation analysis of *ompk35/36* and expression analysis of *bla*_KPC-2_.

**FIG 2:**
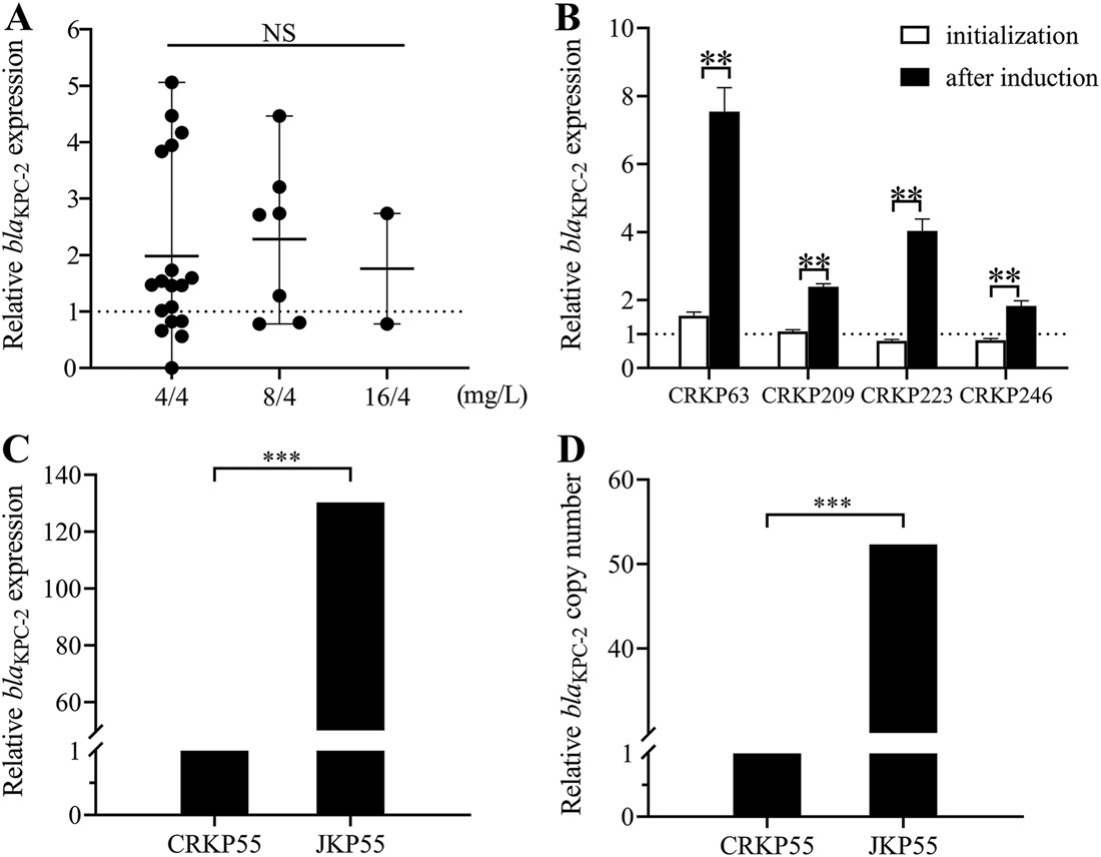
Relative expression and copy number of the *bla*_KPC-2_ gene, expressed as fold change in selected isolates. (A) Relative *bla*_KPC-2_ in 25 clinical K. pneumoniae isolates. Unpaired *t* test with Welch’s correction. (B) Relative *bla*_KPC-2_ expressions in origin strains and induced resistant strains. **, *P* < 0.05; paired *t* test. Klebsiella pneumoniae BAA-1705 was used as the reference strain (expression = 1), which is indicated by black dotted horizontal lines. (C) The relative copy number of the *bla*_KPC-2_ gene in JKP55 was 130-fold higher than that in the KP55 strain. (D) The copy number of the *bla*_KPC-2_ gene in JKP55 was 52-fold higher than that in KP55. Data were evaluated using paired *t* tests. The statistical software used in this study was GraphPad Prism 8. ***, *P* < 0.005.

No mutation of *bla*_KPC-2_ was observed in the 25 CRKP isolates. However, among the induced-resistance strains, a mutation predicted to encode R164S was observed in YKP209, and a mutation predicted to encode D176N was observed in JKP246 (Table 1). Sequencing of the outer membrane porin genes *ompK35* and *ompK36* showed that 25 isolates contained a mutant *ompK35*, 24/25 strains and one strain had a premature stop codon at amino acid position 63 and amino acid position 82, respectively. Additionally, 24 of the 25 isolates had alterations in the L3 region of OmpK36 owing to glycine and aspartic acid duplication at amino acid 135 (134 to 135 GD insertion), and only one strain had a frameshift mutation at amino acid 184 (Fig. 1). With the *bla*_KPC-2_ expression value of the standard strain BAA-1705 used as a reference, no significant difference in *bla*_KPC-2_ expression level was observed between the 4/4-mg/L strains, 8/4-mg/L strains, and BAA-1705. However, compared to the initial strains, the expression of *bla*_KPC-2_ in the induced-resistant strains increased to different degrees. Among them, YKP63 and YKP223 showed a 4.9-fold increase compared with the original strains, and YKP223 and YKP246 showed a 2.2-fold increase (Fig. 2A). Surprisingly, the relative expression of *bla*_KPC-2_ in the conjugant JKP55 was nearly 130-fold higher than that in the donor strain CRKP55 (Fig. 2C).

### Plasmid sequencing and analysis.

**FIG 3:**
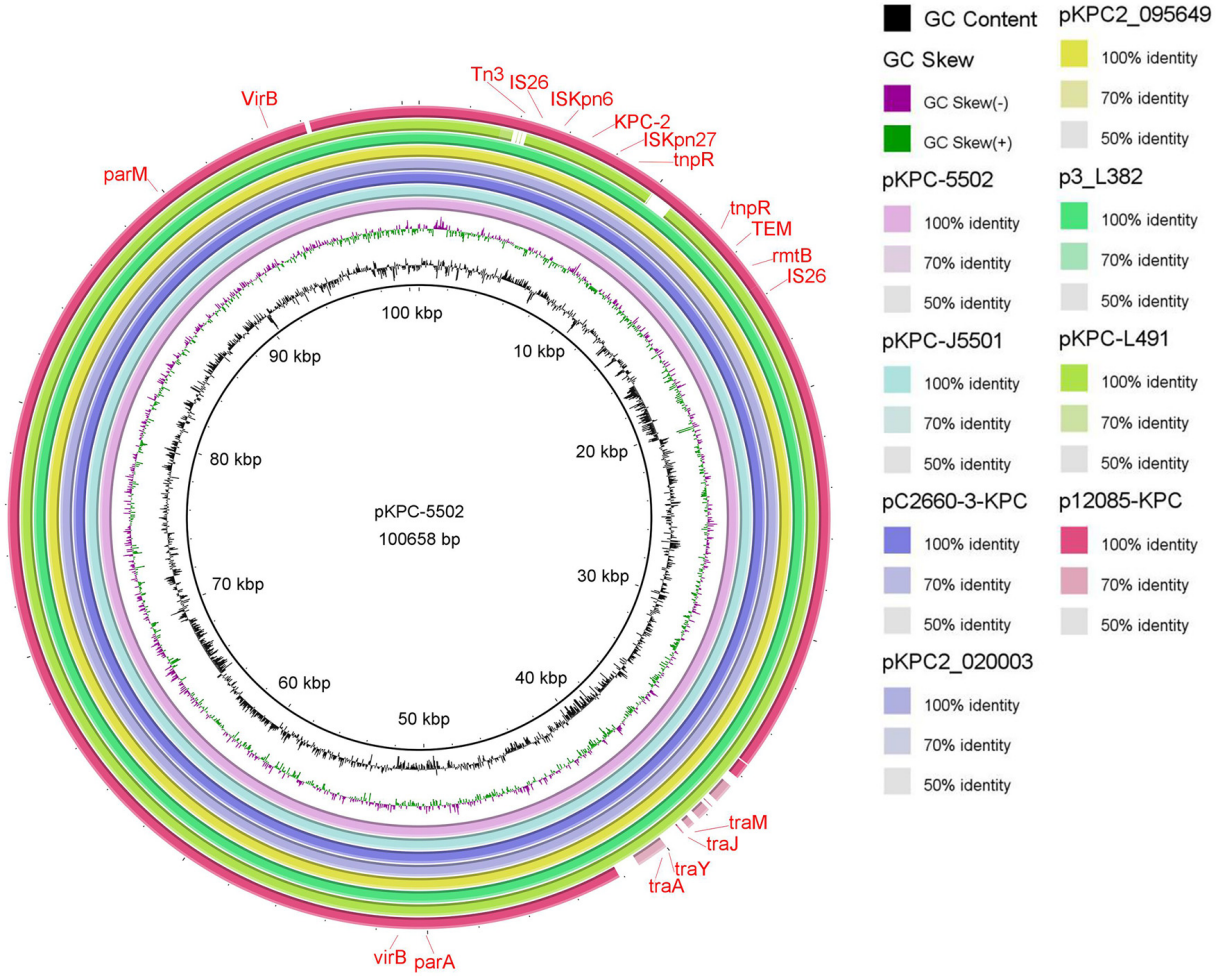
Alignment of plasmids. Comparison of the plasmids pKPC-5502, pKPC-J5501, and other plasmids using BLAST Ring Image Generator (BRIG). A BLAST search for the contig in GenBank showed that the sequence of pKPC-5502 was very similar (99.97% coverage and 100% identity) to those of pC2660-3-KPC (153,556 bp, GenBank accession no. NZ_CP039810), a plasmid of K. pneumoniae isolated in Beijing, China, pKPC2_020003 (154,957 bp, accession no. NZ_CP031720), a plasmid of K. pneumoniae isolated in Chengdu, China, p3_L382 (136,159 bp, accession no. NZ_CP033962), a plasmid of K. pneumoniae isolated in Guangzhou, China.

**FIG 4:**
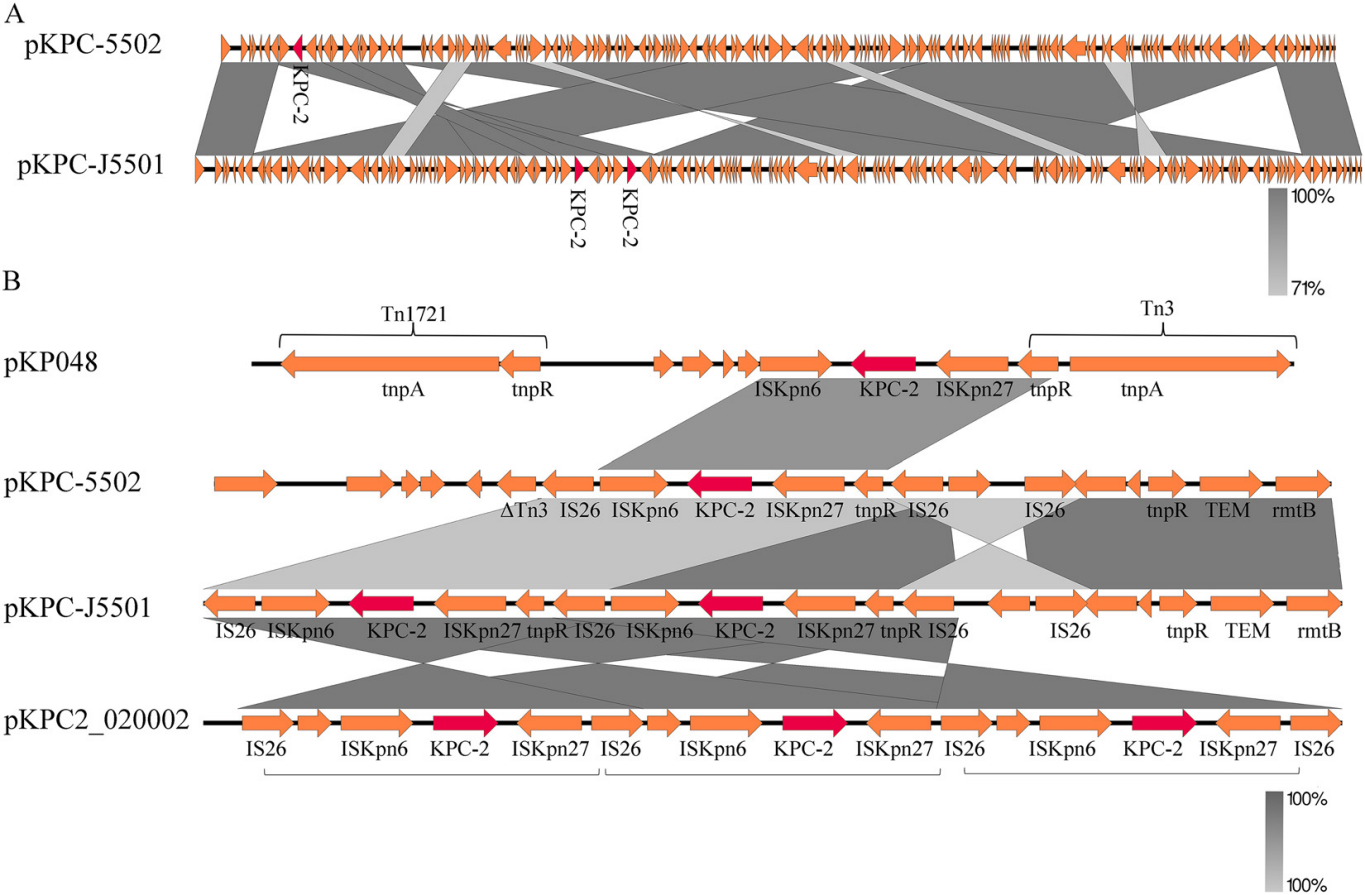
Genetic features of pKPC-5502 and pKPC-J5501. (A) Linear sequence comparison of pKPC-5502 and pKPC-J5501. (B) Genetic environment of *bla*_KPC-2_ in plasmids pKPC-5502 and pKPC-J5501 and comparison of the structures with plasmid pKP048 (GenBank accession no. FJ628167) and pKPC2_02002 (GenBank accession no. NZ_CP028541). The plasmid pKP048, found in China, contained a classic *bla*_KPC-2_ genetic environment, including a Tn*1721*-*bla*_KPC_-Tn*3*-like structure. The *bla*_KPC-2_ gene is marked with red.

Five plasmids, pKPC-5501, pKPC-5502, pKPC-5503, pKPC-5504, and pKPC-5505, existed in CRKP55, while there was only one plasmid, pKPC-J5501, in JKP55. The plasmid pKPC-5501 (231,223 bp) contained an IncFI(K) and an IncFII(K) replicon, with the virulence genes *iutA*, *iucC*, and *traT* on it. The plasmid pKPC-5502 (100,658 bp), an IncFII (pHN7A8) replicon, carried the insertion sequence (IS)*26*-mediated antimicrobial resistance genes (ARGs) (*bla*_KPC-2_, *bla*_TEM_, and *bla*_rmtB_). A Basic Local Alignment Search Tool (BLAST) search of the contig in GenBank showed that the sequence of pKPC-5502 was very similar (99.97% coverage and 100% identity) to those of pC2660-3-KPC (153,556 bp, GenBank accession no. NZ_CP039810), a plasmid of K. pneumoniae isolated from Beijing, China, pKPC2_020003 (154,957 bp, accession no. NZ_CP031720), a plasmid from K. pneumoniae isolated in Chengdu, China, and p3_L382 (136159 bp, accession no. NZ_CP033962), a plasmid of K. pneumoniae isolated in Guangzhou, China (Fig. 3). Linear sequence comparison of the plasmids pKPC-5502 and pKPC-J5501 suggested that there might be a rearrangement during conjunction (Fig. 4A). The genetic structure of *bla*_KPC-2_ in pKPC-5502 is different from that of pKP048 (10), which has a *bla*_KPC-2_ genetic structure (Tn*1721*-*bla*_KPC_-Tn*3*-like structure) in China, carrying an IS*26*-based composite transposon. In pKPC-5502, a 5.5-kb region including *bla*_KPC-2_ and two flanking IS*26* elements resembled a composite transposon, with basic linear structure IS*26-*tnpR-IS*Kpn27-bla*_KPC-2_-IS*Kpn6-*IS*26*. Surprisingly, there were two copies of *bla*_KPC-2_ on pKPC-J5501, and each of the *bla*_KPC-2_ genes was located in the same genetic context as pKPC2-020002 (11), a plasmid with three copies of the IS*26*-IS*Kpn27*-*bla*_KPC-2_-IS*Kpn6*-IS*26* unit (Fig. 4). Furthermore, there were no significant differences in the growth curves between JKP55 and EC600 (Fig. S1), indicating that the plasmid pKPC-J5501 had no fitness costs.

## DISCUSSION

K. pneumoniae has accumulated a wide range of resistance determinants and has evolved into a difficult-to-treat pathogen that poses an increasing threat to health care. KPC is an important marker for extensively drug-resistant organisms with limited treatment options. CRKP strains are highly resistant to cephalosporins, carbapenems, aminoglycosides, and fluoroquinolones, making colistin and tigecycline the last-line drugs. CAZ/AVI has good activity against CRKP and offers a significant advantage over colistin, gentamicin, and tigecycline, which are limited by concerns over efficacy and/or toxicity. With the increasing incidence of KPC infections worldwide, the emergence of CAZ/AVI undoubtedly brings hope to the treatment of CRKP. However, there have been many reports about CAZ/AVI resistance in KPC-producing strains since the approval of CAZ/AVI.

Among 127 CRKPs collected from 110 patients in our study, 16 strains showed resistance to CAZ/AVI (12.6%), which was lower than the CHINET 2018 Surveillance result (15.4% [12] in CRKPs). Multifactorial analysis was performed in these strains, and it was found that the use of cephalosporin antibiotics was related to CAZ/AVI sensitivity (Table S2). In seven patients, the sensitivity to CAZ/AVI changed after cephalosporin or MEM administration from reduced susceptibility to susceptibility. Some interesting results were found for the three CRKPs isolated from the same patient. The first isolate showed reduced susceptibility to CAZ/AVI when sulbactam and cefoperazone were administered to the patient, while the second isolate recovered susceptibility. However, after the discontinuation of imipenem, the strain became resistant to both CAZ/AVI and MEM. The evolutionary trajectory of drug resistance in these strains requires further investigation.

In addition, we found 25 strains with decreased susceptibility to CAZ/AVI that did not produce MBL, and all but 2 isolates were below the CLSI resistance breakpoint. To investigate the mechanism of the decreased sensitivity, we evaluated the expression of *bla*_KPC-2_ and successfully induced four resistant strains. There was no significant difference in the expression levels of *bla*_KPC_ between the reduced susceptibility strains and BAA-1705, which differed from the results of Cui et al. (13). The limited number of reduced-susceptibility strains may explain why our results were different. Previously reported gene mutations related to CAZ/AVI resistance (e.g., D179Y) were absent in the 25 strains (14). Premature termination of OmpK35-encoding genes is a common characteristic of clinical isolates of extended-spectrum β-lactamase (ESBL)-producing K. pneumoniae (15). In a study by Tsai et al. (16), K. pneumoniae isolates harboring a deletion of *ompK36* or both *ompK35* and *ompK36* were more resistant to ceftazidime than those of the wild type or a construct carrying only an *ompK36* deletion. Subsequent studies had also found that the mutant *ompK35* (premature termination) and *ompK36* (134 to 135 GD insertion) were related to reduced susceptibility to CAZ/AVI (17–19). Combined with our current results, we speculate that perhaps it is the deficiency of outer membrane proteins that results in the diminished penetration of ceftazidime through OmpK35/36 porins in the presence of ESBL and KPC enzymes with good hydrolytic profiles for ceftazidime, which could be the reason for the elevated CAZ/AVI MIC results in our study.

Mutations in the 164 to 179 site of the Ω-loop of KPC decreased the binding of AVI, leading to CAZ/AVI resistance. We found that the induced resistance strains YKP209 and YKP246 had substitutions in the KPC-2 Ω-loop (R164S and D176N, respectively). All the induced resistant strains had increased expression of *bla*_KPC-2_, suggesting that the overexpression and mutation of *bla*_KPC-2_ may be responsible for the change in resistance.

Similar to other regions in China (20), the ST11 clone was the most prevalent strain type in our hospital, accounting for as much as 82.7% (105/127) of the carbapenemase-producing K. pneumoniae strains tested in this study, followed by the ST1887 clone. All strains with reduced sensitivity belonged to the ST11 clone, and ERIC divided these 25 strains into 4 clusters. CRKP55 belongs to the most abundant cluster, and the pKPC-5502 plasmid it carries is similar to the IncFII plasmid isolated from K. pneumoniae in Beijing, Chengdu, Guangzhou, and other locations, suggesting that the IncFII plasmid with such a backbone structure is widespread in China and may play an important part in the spread of *bla*_KPC-2_ resistance genes.

It is well known that two copies of IS*26* can form a composite transposon, which may be excised from plasmids to form a translocatable unit (TU) (21–23). IS*26* provides a region for homologous recombination and could therefore serve as a Trojan horse in the presence of IS*26* on a plasmid, which may be integrated into the plasmid via homologous recombination to generate tandem repeats (24). Previous studies have shown that IS*26* is involved in the dissemination and amplification of *bla*_KPC-2_ (25). Double copies of *bla*_KPC-2_ were observed in pKPC-J5501, and *bla*_KPC-2_ was flanked by IS*26*. Therefore, we hypothesized that the double copies were mediated by IS*26* homologous recombination. In addition, the copy number of plasmid pKPC-J5501 was greatly increased owing to the low pressure in EC600, resulting in a large increase in the expression of *bla*_KPC-2_. In summary, CAZ/AVI resistance in JKP55 is related to the overexpression of *bla*_KPC-2_, and IS*26*-mediated gene amplification contributed to the elevation of CAZ/AVI MIC.

In conclusion, the comparison of expression levels of 25 reduced-sensitivity isolates suggested that there may be no significant correlation between expression and reduced sensitivity; however, under the condition of antibiotic pressure, they could evolve resistance due to the increased expression level or substitutions in the Ω-loop of KPC-2. In addition, it is speculated that the *bla*_KPC-2_ gene located in the conjugative plasmid can undergo tandem amplification during horizontal transfer from strain to strain, resulting in increased resistance to CAZ/AVI.

## MATERIALS AND METHODS

### Bacterial isolates and confirmation of carbapenemase production.

CRE isolates were defined as isolates which were resistant to one or more carbapenems tested in our institution (i.e., ertapenem, imipenem, or MEM) according to CLSI (Clinical and Laboratory Standards Institute) breakpoints or produced carbapenemases. A total of 331 nonduplicated CRE strains were collected and identified using a Vitek-2 automated microbiology analyzer (bioMérieux, France) from 2018 to 2020. A total of 127 CRKP strains were used to conduct further research. The modified carbapenem inactivation method (mCIM) and EDTA-modified carbapenem inactivation method (eCIM) were used to screen carbapenemase production as previously described (26). mCIM and eCIM were performed according to document M100-S27 of the CLSI. Briefly, a 1-μL loopful of CRKP colonies was inoculated into 2-mL aliquots of tryptic soy broth (TSB). The suspension was vortexed and a 10-μg MEM disk (Oxoid) was placed in each inoculation tube. The tubes were incubated for 4 h (±15 min). Then, the MEM disk was removed from the inoculated tube and attached to the Mueller-Hinton agar (MHA) plate with the ATCC 25922 strain. For eCIM testing, each isolate was inoculated in a 2-mL aliquot of TSB-EDTA (EDTA concentration, 5 mM). Isolates were incubated and plated as described for mCIM testing. Plates were incubated for 18 to 24 h, and the interpretation for both assays was done according to CLSI-2020. Common carbapenemase genes, including *bla*_KPC_ and *bla*_NDM_, ESBLs, and other resistance genes (e.g., *bla*_IMP-4_, *bla*_IMP-8_, *qnrA*, and *qnrB*) were identified by PCR with primers as reported previously (27). Genomic DNA used as PCR template DNA was extracted from each CRKP sample by the boiling method. Amplicons were visualized after electrophoresis at 85 V for 50 min on a 1.2% agarose gel, positive amplification products were subjected to Sanger sequencing, and the sequencing results were compared using BLAST. All isolates were subjected to multilocus sequence typing (MLST) and ERIC-PCR.

### Determination of CAZ/AVI MICs.

To determine the antibiotic susceptibility of the 127 strains, MICs were determined using the broth microdilution method, according to the protocol of the CLSI. For antibiotics including imipenem, MEM, amikacin, levofloxacin, and polymyxin, Escherichia coli ATCC 25922 was used as a quality control strain, and the MIC values were interpreted according to CLSI breakpoints. For CAZ/AVI, E. coli ATCC 25922 and K. pneumoniae ATCC 700603 were used as the quality control strains. Avibactam was tested at a fixed concentration of 4 mg/L, in combination with 2-fold dilutions of ceftazidime. MICs were interpreted according to the CLSI susceptible breakpoint of ≤8/4 mg/L. As previously described (13, 17), a CAZ/AVI MIC of ≥4/4 mg/L was used as the cutoff for reduced susceptibility to CAZ/AVI in this study.

### *In vitro* induction test.

The 23 strains that did not produce MBL showed reduced susceptibility to CAZ/AVI but were not resistant to it, and none of these strains had an exposure history to CAZ/AVI (no relevant antibiotic use record in the patients’ hospitalization records). To detect changes in bacterial resistance under CAZ/AVI selective pressure conditions, we conducted an *in vitro* induction test using a previously described method (13). A total of 8 of the strains (4 strains selected from 18 CRKP isolates with a MIC of 4 mg/L and 4 strains selected from 5 CRKP isolates with a MIC of 8 mg/L) were randomly selected and cultured overnight in 1 mL Luria-Bertani (LB) broth supplemented with CAZ/AVI concentration at 0.25 fold MICs of each strains. Positive bacterial growth was further incubated with a 2-fold-increased concentration of CAZ/AVI. This procedure was repeated daily until these strains reached a CAZ/AVI concentration of 128/4 mg/L or a CAZ/AVI concentration that did not allow bacterial growth. The broth microdilution method was used to detect the MIC values of CAZ/AVI for these isolates, and isolates with a MIC of ≥16 mg/L after CAZ/AVI selection were defined as induced resistant strains. Corresponding isolates that grew in antibiotic-free tubes were used as positive controls, and the ATCC 25922 strain grown in the tubes with corresponding concentration of CAZ/AVI was used as the negative control.

### Conjugation experiment and growth assay.

To assess whether *bla*_KPC-2_ genes were located on plasmids and the transferability of plasmids, 25 strains were conjugated with E. coli EC600 (for strains susceptible to CAZ/AVI, no conjugation experiment was carried out). The conjugation experiment was performed using a membrane bonding experiment, as previously described (26). Both the donor (CRKPs) and recipient strain (E. coli EC600) were mixed in LB broth at a ratio of 1:3, and the mixtures were placed on a membrane and incubated for 24 h at 35°C. The transconjugants were selected on MHA plates supplemented with rifampicin (600 mg/L) and MEM (1 mg/L). EC600 and donor strains cannot both grow on plates supplemented with rifampicin (600 mg/L) and MEM (1 mg/L), which was used as the negative control for plasmid conjugation experiments. Strains can grow on plates without antibiotic, which were used as positive controls. Colonies that grew on the selective medium were identified using the Vitek-2 compact system and 16S rRNA sequencing. 16S rRNA primers was used to detect the 16S rRNA gene through PCR. The positive products were subjected to Sanger sequencing, and the sequences were then subjected to BLAST search with sequences in NCBI to identify the isolates and thus distinguish them from the donor strains. Antimicrobial susceptibility testing was performed to detect changes in the resistance of conjugants using the broth microdilution method. A growth assay was performed to assess the fitness costs of JKP55 and EC600. The growth assay was performed as previously described (28). Overnight cultures from single colonies of JKP55 and E. coli EC600 were adjusted to 0.5 McFarland standard. Then, 5 μL of the adjusted cultures were diluted into 5 mL using LB broth and incubated at 37°C and 200 rpm for 24 h. Bacterial growth was monitored by measuring the optical density at 630 nm (OD_630_) using 96-well plates every 2 h for 24 h at 37°C. The experiments were performed in triplicate.

### Mutation and expression analysis of *bla*_KPC-2_, *ompK35*, and *ompK36*.

PCR was used to amplify the full-length *bla*_KPC-2_, *ompk35*, and *ompk36* genes. The Sanger sequencing results were compared with those of standard sequences. Total RNA was extracted from overnight cultures using a Pure Link RNA minikit (Thermo Fisher Scientific, USA), according to the manufacturer’s instructions. cDNA was obtained using a PrimeScript real-time (RT) reagent kit with gDNA Eraser (TaKaRa, Kyoto, Japan). Quantitative PCR (qPCR) was performed using TB Green premix *Ex Taq* (TaKaRa, Kyoto, Japan) on the CFX96 real-time PCR system. Relative gene expression levels were calculated using the 2^–ΔΔ^*^CT^* formula with the *rpoB* gene as the internal control. All samples were analyzed in triplicate. The *bla*_KPC-2_ expression level in the BAA-1705 strain was used as a reference.

### Plasmid sequencing and analysis.

Plasmids in CRKP55 and its conjugant JKP55 were sequenced using an Illumina HiSeq PE150 instrument, and the assembly was annotated using Prokka v1.12. The resistance genes were predicted by Resistance Gene Identifier (RGI) v5.1.1 in the Comprehensive Antibiotic Resistance Database (CARD) platform (https://card.mcmaster.ca/). Insertion sequences (ISs) were identified using ISfinder (https://www-is.biotoul.fr/) and TnCentral (https://tncentral.proteininformationresource.org/). Plasmid sequences were compared using BLAST Ring Image Generator (BRIG) v0.95. The genetic environment of *bla_KPC-2_* was generated using Easyfig 2v.2.

### Data availability.

Plasmids sequence data have been deposited in NCBI. The nucleotide sequences of the pKPC-5501, pKPC-5502, pKPC-5504, pKPC-5505, and pKPC-J5501 plasmids have been deposited in GenBank under accession numbers OL891651, OL891652, OL891654, OL891655, and OL891656, respectively.
