# Supplementary material for: Increased Expression and Amplification of blaKPC-2 Contributes to Resistance to Ceftazidime/Avibactam in a Sequence Type 11 Carbapenem-Resistant Klebsiella pneumoniae Strain
Source: Microbiol Spectr. 2022 Jul 28;10(4):e00955-22. doi: 10.1128/spectrum.00955-22 (PMC9430841; doi:10.1128/spectrum.00955-22)
Supplement: Supplemental file 1 — Table S1, Table S2, Fig. S1. Download spectrum.00955-22-s0001.pdf, PDF file, 0.2 MB [file spectrum.00955-22-s0001.pdf]

Table S1. MICs change of CAZ/AVI and resistant characteristics during the conjugation experiment.

Isolates	MIC (mg/L)				Resistance genes
	IPM	MEM	CAZ/AVI	PB	
KP55	64	512	4/4	4	KPC, TEM, SHV
KP58	64	512	4/4	4	KPC, TEM, SHV
KP63	256	256	4/4	2	KPC, SHV
KP232	64	>128	4/4	2	KPC, TEM
KP314	64	>128	4/4	2	KPC, TEM
KP318	64	>128	4/4	2	KPC, TEM
Conjugations					
JKP55	4	16	⩾256/4	1	KPC, TEM
JKP58	16	16	1/4	0.5	KPC, TEM
JKP63	4	4	0.25/4	0.5	KPC
JKP232	8	32	0.25/4	1	KPC, TEM
JKP314	8	16	0.25/4	1	KPC, TEM
JKP318	2	16	0.25/4	1	KPC, TEM

Table S2. Multifactor analysis of antibiotic resistance to CAZ/AVI.

Related factors	Group	susceptibility to CAZ/AVI(CRKPs)						X^2/Z	P
		susceptible		reduced susceptibility		Resistance			
Cephalosporin [a]	Use	35		14		11		8.252	0.015
	Non-use	53		11		3			
Carbapenem [b]	Use	56		16		5		4.093	0.142
	Non-use	32		9		9			
Invasive procedure [a]	Implemented	53		21		5		17.238	0.000
	None	19		1		9			
Length of stay [b]	<14day	13		4		6		4.055	0.157
	≥14day	59		20		8			
		ICU	Other	ICU	Other	ICU	Other		
Clinical outcome [b]	Survival	30	35	22	0	22	0	0.979	0.763
	Death	6	1	2	0	2	0		

[a] represented $P < 0.05$, [b]represented $P > 0.05$. susceptible group: non-MBL-producing strains, susceptible to CAZ/AVI. reduced susceptibility group: non-MBL-producing strains, reduced susceptibility to CAZ/AVI. resistance group: resistance to CAZ/AVI, producing NDM.

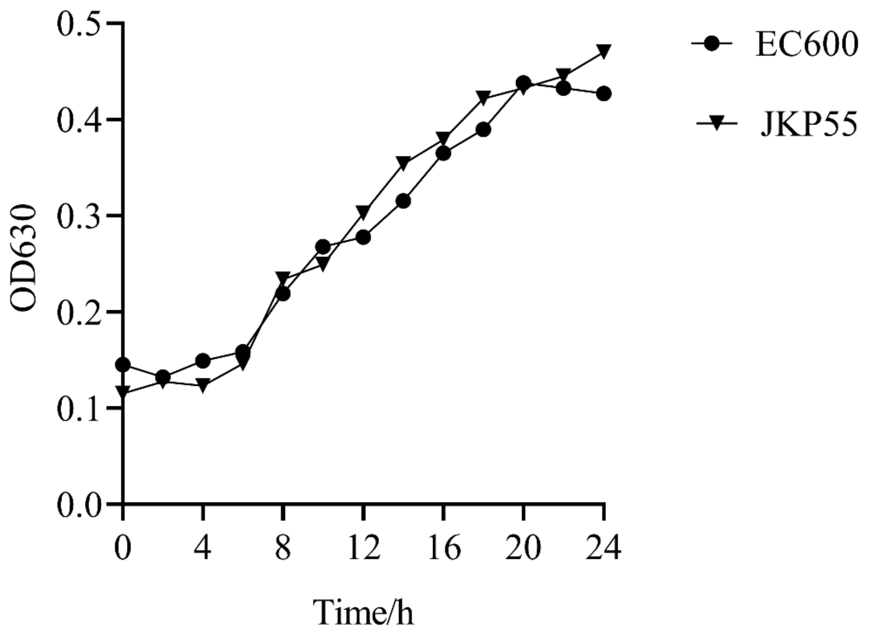

Figure S1. Growth curves of JKP55 and EC600. P=0.4143. There were no significant differences between the two strains.
